# Predictors of Duration of Transjugular Intrahepatic Portosystemic Shunt (TIPS) Procedure: A Retrospective Single-Center Study

**DOI:** 10.7759/cureus.65776

**Published:** 2024-07-30

**Authors:** Juan Carlos Barrera Gutierrez, Melissa Zullo, Seth Sclair, Sidhartha Tavri

**Affiliations:** 1 Interventional Radiology, University Hospitals Cleveland Medical Center, Case Western Reserve University, Cleveland, USA; 2 Methodist Digestive Institute, Methodist Health System, Dallas, USA; 3 College of Public Health, Kent State University, Kent, USA; 4 Gastroenterology and Hepatology, University Hospitals Cleveland Medical Center, Cleveland, USA; 5 Interventional Radiology, University Hospitals Cleveland Medical Center, Cleveland, USA

**Keywords:** portal hypertension, cirrhosis, liver fibrosis, duration of the tips procedure, predictors, transjugular intrahepatic portosystemic shunt

## Abstract

Purpose: To determine the relationship between clinical, procedural, hospital, and physician characteristics with the duration of the transjugular intrahepatic portosystemic shunt (TIPS) procedure.

Methods: This retrospective study included patients over 18 years of age who underwent an initial TIPS procedure between January 2005 and August 2020. Exclusion criteria were TIPS performed outside the institution and failed TIPS placement. A total of 154 records were included. Regression analyses were used to identify predictors of procedural duration.

Results: The mean age at TIPS placement was 57 years. Seventy percent of patients were male and non-Hispanic whites (80.5%). The mean duration of the TIPS procedure was 169 minutes (SD: 78). Procedural duration was shorter when the etiology of cirrhosis was viral (mean: 144 min, SD: 84, p=0.008); the reason for TIPS was ascites (152, SD: 66, p=0.01); and the procedure did not require additional access (153 min, SD: 67, p=<.0001). The main clinical predictor of procedural duration was baseline bilirubin (Beta coefficient (β): 5.6 min, p=0.0007). In multivariable linear models, in those patients that did not require additional access, bilirubin (β: 4.9 min, p=0.005), etiology of cirrhosis, and physician experience were the main predictors of TIPS procedure duration. The effect of baseline bilirubin on procedural duration increased in the ascites group (β: 19.5 minutes, p=0.006), especially when additional access was not required.

Conclusions: The study demonstrates an association between baseline bilirubin, etiology of cirrhosis, and physician experience with the duration of the TIPS procedure. The mechanism underlying the positive association between baseline bilirubin and procedural time is possibly related to the degree of liver fibrosis.

## Introduction

Transjugular intrahepatic portosystemic shunt (TIPS) involves a communication through a stent graft between the portal vein system and the hepatic veins. Its purpose is to decrease the pressure in the portal vein system, which is often caused by cirrhosis. TIPS is a challenging intervention procedure that requires a skilled interventional radiologist (IR) physician, and relatively high radiation doses (mean cumulative air kerma > 1.7 Gy) [1]. A single TIPS procedure may require several needle liver punctures to establish the communication with the portal vein, and the range of passes may vary from one to 35 [2,3]. Each puncture attempt increases the procedural time. Needle punctures can cause hepatic artery injury, biliary puncture or transection with significant morbidity and mortality [3].

Patients' characteristics can make surgical procedures more challenging and even reduce IR interventions' efficacy. For example, obesity can increase the risk of certain complications, such as hypertension or wound infection, during and after surgery. Patients with cirrhosis frequently present with obesity, severe ascites, and small cirrhotic livers. These conditions can attenuate the imaging modalities used to identify anatomical structures, thus requiring the IR physician to modify computerized tomography (CT) scan parameters, or use other strategies such as percutaneous access, or additional imaging modalities, such as intravascular ultrasound [4]. Moreover, large ascites can make fluoroscopic visualization more difficult, leading to increased radiation exposure [5]. All these strategies or imaging modalities tend to prolong the duration of a TIPS procedure.

Physician experience has been used to predict duration of surgical interventions [6]. Time-consuming operations have an impact on cost, hospital efficiency, and patient health safety. Furthermore, hospitals that perform a high volume of surgical procedures achieve better outcomes [6,7].

The impact of increased duration of the TIPS procedure and the factors that can affect the procedure's duration have not been evaluated. The purpose of this study was to determine the relationship between patient characteristics, procedural, staff factors, and type of hospital with the duration of the TIPS procedure. In this study duration of TIPS procedure was used as a surrogate of number of needle punctures required to get the shunt, the more passes or punctures required for communicating the hepatic vein with the portal vein, the longer the procedure.

## Materials and methods

Study design and patient population

Institutional Review Board approval was obtained for this retrospective single-center study (approval: approval STUDY20200281). Data collection was performed manually from electronic medical records. The REDcap system was utilized for data tabulation and organization. Inclusion criteria were patients over 18 years of age who underwent an initial TIPS procedure between January 2005 and August 2020. Exclusion criteria were those TIPS performed outside of the institution and failed procedures.

The study used a convenience sample of 165 medical records identified with the diagnosis of TIPS in the billing process. Eleven patients were excluded, 10 due to failure to access the portal vein, and one patient who was incorrectly identified as TIPS. In total, the study included 154 patients.

Predictors: demographic, clinical, procedure, and staff characteristics

Demographics and Patient Clinical Variables

The collected variables included: age at the time of the procedure, sex, race/ethnicity which was classified into three categories (non-Hispanic Whites, non-Hispanic Blacks, Hispanics and other ethnicities), and date of TIPS procedure. The patient’s clinical characteristics included: anthropometric measures such as height (in centimeters), and weight (in kilograms). Body mass index (BMI) was calculated from weight and height using the formula (weight [kg]/ height [m]2), and categorized in three levels: BMI <25, BMI ≥ 25 and < 30, and BMI ≥30. Etiology of cirrhosis was categorized as viral, alcohol-related, non-alcoholic steatohepatitis (NASH), and other etiologies. Smoking status and drinking status were classified as current, former, and never smoker or never drinker.

Patient comorbidities included hypertension, chronic obstructive pulmonary disease (COPD), diabetes mellitus, hypothyroidism, and cancer. Cardiovascular disease (CVD) was included if any one of the following diagnoses was documented in the record: stroke, myocardial infarction, or coronary artery disease. Chronic kidney disease was defined as present if the baseline serum creatinine was greater or equal to 1.5 mg/dL (normal value 0.7- 1.4mg/dL) [8]. Symptoms and complications associated with portal hypertension included ascites, hydrothorax, gastroesophageal (GE) varices (including ectopic varices and portal hypertensive gastropathy), hepatic encephalopathy, and hepatorenal syndrome. These symptoms were dichotomized into present or absent if they were described in the medical records or their presence was clearly depicted in any of the associated imaging procedures, such as abdominal ultrasound, abdomen CT scan, chest X-ray, or esophagogastroduodenoscopy.

Laboratory baseline measurements were obtained from pre-surgical evaluation within the previous week and/or as close to the day of the procedure as possible. These labs included: hemoglobin (Hb, gr/dl) and serum creatinine (sCr, mg/dL). Liver function tests (LFTs) included serum bilirubin (mg/dL), alanine transaminase (ALT, mg/dL), aspartate transaminase (AST, mg/dL), alkaline phosphatase (ALP, mg/dL), and international normalized ratio (INR). Given the better ability of Model for End-stage Liver Disease (MELD) score to predict 30 days mortality than MELD sodium [9], the MELD score was recalculated using the following formula: MELD score = 9.6 loge (creatinine mg/dl) +3.8 loge (bilirubin mg/dl) +11.2 loge (INR) +6.4 (cause of cirrhosis [0 if cholestasis or alcoholic, 1 otherwise]). Scores ranged from 6 to 40.

Variables Related to the Procedure

Procedural-related factors included: ‘diameter of portal vein’, obtained during the pre-surgical Doppler ultrasound evaluation (measured in millimeters). ‘Indications for TIPS placement’ included: acute variceal bleeding, gastroesophageal or ectopic varices, ascites, hydrothorax, surgery or pre-transplant, and other indications. The ‘procedure type’ was classified as an elective or emergency procedure when the patient was active bleeding. The ‘reason for the TIPS’ procedure was divided into ascites (when the indication of TIPS was ascites, hydrothorax, or surgery/pre-transplant) and gastrointestinal (GI) bleeding (included acute variceal bleeding, and gastroesophageal varices). The variable ‘Access’ was categorized in ‘additional accesses or procedures’ when the patient required access for intravascular ultrasound, percutaneous accesses of the portal vein (trans-hepatic or trans-splenic), umbilical access, and variceal embolization. When the internal jugular vein was the only access point, the group was termed ‘No additional accesses.’ Paracentesis was assigned as ‘yes’ if it was performed immediately before the TIPS, otherwise as ‘no’; the volume of ascites removed during paracentesis was quantified in liters.

Variables Related to the Intervention, Healthcare Provider, and Hospital Characteristics

Healthcare provider or interventional radiologist (IR) experience was measured in years for the physician performing the procedure. This variable was obtained by subtracting the year of IR fellowship graduation from the year of the TIPS procedure. IR experience was categorized in three levels: less than two years, two to four years, and five or more years. Type of hospital was classified as ‘major teaching hospital’ or any ‘other hospital’ if it was not considered a teaching institution.

Outcome Variable

The time or duration of the TIPS procedure (procedural time) was recorded in minutes (min), and it was the result of subtracting the starting time from the end time. The starting time was determined using the imaging ultrasound when the physician accessed the patient's internal jugular vein. The end time was the time recorded in the final portal venogram after TIPS insertion.

Data and statistical analysis

The overall missing data was 8%, and missing values for MELD calculation were 2% of sample size. The missing values for the MELD score were imputed with the median of each variable. For statistical calculations, missing data was handled with listwise deletion by the software package. All continuous variables were tested for normality and presented with means, standard deviation, medians, and range (minimum and maximum values). Categorical data were expressed as count and percentage. The t-test and the ANOVA test were used to compare means among the strata of categorical variables. Wilcoxon Mann-Whitney or Kruskal-Wallis tests were utilized in variables non-normally distributed. Procedural time followed a non-normal distribution; however, did not require any transformation according to the sample size, skewness, standard error (se), and Z score (Z = skew/se, not requiring transformation if Z < 3.2) [10]. Simple linear regression (SLR) analysis was used to establish the association between the continuous predictors and duration of the TIPS procedure. Forward variable selection was adopted to build the final regression models, including predictors with a significant level ≤ 0.2. Variables with p ≤ 0.05 were kept in the final models. The interaction between a third variable and the control variable was tested to rule out that the effect on the outcome did not depend on this interaction. SAS version 9.4 (SAS Institute Inc., Cary, NC, USA) was used for all analyses; two-tailed p < 0.05 was considered statistically significant.

## Results

Baseline characteristics

The mean (SD) age at the TIPS procedure was 57 (SD: 11.6) years. Among the patients, 70% (108/154) were male. Non-Hispanic Whites constituted the largest group at 80.5% (112/154), followed by African Americans at 15% (21/154). Forty percent (57/154) of patients were classified with a BMI > 30. Alcohol was the primary etiology of cirrhosis in 44% (68/154), followed by viral hepatitis C in 28% (43/154). The main symptoms of portal hypertension were ascites in 88% (135/154) and GE varices in 80% (122/154), followed by hepatic encephalopathy in 19% (29/154). Hypertension was the most frequent comorbidity, present in 55% of individuals (84/154), followed by diabetes in 41% (63/154). For more information, refer to Table 1.

**Table 1:** Demographic, Clinical, Procedural, Hospital, and Healthcare Provider Characteristics of TIPS procedures, n=154 Abbreviations: N: number, SD: standard deviation, cm: centimeter, Kg: kilogram, NH: non-Hispanic, BMI: body mass index, NASH: non-alcoholic steatohepatitis, GE: gastroesophageal, HRS: hepatorenal syndrome, COPD: chronic obstructive pulmonary disease, GI: gastrointestinal, IR: interventional radiologist, TIPS: transjugular intrahepatic portosystemic shunt

Variable	number	percentage
Age, mean, and SD	56.9	11.6
Sex		
Females	46	30
Males	108	70
Height cm. mean, and SD	173	11
Weight kg. mean, and SD	87	24
BMI categories		
BMI<25	34	24
BMI≥25< 30	50	36
BMI>30	57	40
Race/ethnicity		
Whites NH	112	81
Black NH	21	15
Hispanic/Other	6	4
Etiology of Cirrhosis		
Alcoholic	68	44
Viral	43	28
NASH	20	13
Other type	22	14
Portal Hypertension Symptoms		
Ascites	135	88
GE varices	122	80
Hepatic encephalopathy	29	19
Hydrothorax	20	13
HRS	4	3
Comorbidities		
Hypertension	84	55
Diabetes	63	41
Cardiovascular disease	41	27
Chronic kidney disease	30	20
COPD	29	19
Cancer	17	11
Hypothyroidism	24	16
Other	65	46
Alcohol use		
Current	51	35
Past	57	40
Never	36	25
Tobacco use		
Current	36	30
Past	36	31
Never	46	39
Procedural, Hospital, and Staff Characteristics		
Indications for TIPS		
Ascites	70	45
GE varices	10	6
Acute GI bleeding	68	44
Hydrothorax	3	2
Surgery	3	2
Reason for TIPS		
Ascites	76	49
GI bleeding	78	51
Type of TIPS		
Elective	86	56
Emergency	68	44
Access: additional no	115	75
Access: additional yes	39	25
Paracentesis, yes	69	45
Litters, mean and SD.	5.5	2.8
Type of Hospital		
Teaching hospital	133	86
Other hospital	21	14
IR Experience categories		
Less than 2 years	42	27
2 to 4 years	45	29
5 or more years	67	44
IR Experience in years		
Mean experience in years, mean, and SD	5.5	5.2

The most common indication to perform a TIPS procedure was GI bleeding in 51% of patients. In 56% of the cases TIPS was an elective procedure. Seventy-five percent of the patients (n=115) did not require additional access or procedures. Among those patients who required additional accesses (n=39), 21 required variceal embolization, 10 needed intravascular ultrasounds, 10 required access through the umbilical vein and/or transhepatic portal access, and one required a direct intrahepatic portocaval shunt. Paracentesis was performed immediately before the TIPS procedure in 45% of the patients, and the mean volume removed was 5.5 liters (SD: 2.8, range: 0.7-12 liters). Eighty-six percent of the procedures were carried out at a major teaching hospital. Finally, IR physicians with five or more years of experience performed 44% of the TIPS procedures.

Table 2 describes the baseline laboratories with means and medians; these values have different clinical meanings because of the non-normal distributions. For example, the mean bilirubin came out to be 2.5 mg/dl, yet the median was 1.2 mg/dl. The liver function tests (AST and ALT) demonstrated similar behavior. The mean MELD score was 15, with a median of 13.

**Table 2:** Baseline Laboratories of Patients Who Underwent TIPS Procedure, n = 154 Abbreviations: SD: standard deviation, mg: milligrams, d/L: deciliter, INR: International Normalized Ratio, AST: aspartate transaminase, ALT: alanine transaminase, ALP: alkaline phosphatase, MELD: Model for End-stage Liver Disease, TIPS: transjugular intrahepatic portosystemic shunt

Variable	Number	Mean	SD	Median	Min -Max
Hemoglobin	153	9.7	2	9.6	5.7-15.4
Creatinine mg/dL	153	1.32	0.7	1.2	0.4-4.0
Sodium mg/dL	152	137	5	138	121-151
Bilirubin mg/dL	151	2.5	3.8	1.2	0.3-24.5
INR	151	1.4	0.3	1.3	0.9-3
AST mg/dL	150	84	213	36	11-1910
ALT mg/dL	151	62	193	29	7-2149
ALP mg/dL	151	122	79	106	21-529
Albumin gr/dL	147	2.7	0.6	2.7	1.5-4.7
MELD	153	15	6	13	6-39

Table 3 shows the TIPS mean procedural time in each one of the categorical variables. Hispanics and other ethnic groups had the most prolonged duration of the TIPS procedure (265 minutes, p=0.07) compared to Whites or African Americans; this difference was not statistically significant. Given the small sample size in the Hispanic and other ethnicities group, and the violation of constant variance assumption, the variable race/ethnicity was excluded from the multiple regression analysis. Patients with cirrhosis of viral etiology had the shortest procedural time (144 min) compared to the other etiologies (191 min), alcoholic etiology (176), and NASH (171 min) (p=0.008). Accordingly with the procedural characteristics, the mean procedural time in patients with GI bleeding was longer when compared with the ascites group (184 min vs. 152, p=0.01), and was longer in patients who required an additional access vs. no additional access (213 min vs. 153, p=<.0001). Although the mean time of the TIPS procedure done by IR physicians with less than two years of experience was longer than that of IR physicians with more than two years of experience, this difference was not statistically significant (p=0.09). The mean procedural time for TIPS was 169 minutes (SD: 78, range: 31-450 minutes).

**Table 3:** Mean TIPS Procedural Time by Demographics, Clinic, Procedural, and Staff Characteristics, n = 154 Abbreviations: SD: standard deviation, NH: non-Hispanic, BMI: Body mass index, NASH: Non-alcoholic steatohepatitis, N: number, GI: gastrointestinal. IR: interventional radiologist, TIPS: transjugular intrahepatic portosystemic shunt

Variable	Mean time	SD	P value
	Minutes		
Sex			
Females	173	75	0.9
Males	167	84	
BMI categories			
BMI<25	161	84	0.6
BMI≥25< 30	176	83	
BMI>30	172	74	
Race/ethnicity			
Whites NH	265	129	0.07
Black NH	152	90	
Hispanic/Other	164	67	
Etiology of Cirrhosis			
Alcoholic	176	76	0.008
Viral	144	84	
NASH	171	68	
Other type	191	68	
Number of Comorbidities			
0-2 comorbidities	170	76	0.8
3 or more comorbidities	166	56	
Alcohol Use			
Current use	166	72	0.15
Past use	157	73	
Never use	191	84	
Tobacco use			
Current use	160	74	0.42
Past use	154	61	
Never use	180	85	
Procedural characteristics			
Reason for Tips			
Ascites	152	66	0.01
GI bleeding	184	85	
Type of tips			
Elective	158	67	0.09
Emergency	181	88	
Paracentesis			
yes	167	77	0.8
no	169	78	
Access			
Additional accesses No	153	67	< .0001>
Additional accesses yes	213	88	
Type of hospital			
Teaching hospital	169	77	0.7
Other hospital	162	79	
IR experience			
Less than 2 years	188	75	0.09
2 to 4 years	164	78	
5 or more years	159	77	

Predictors of procedural time

Table 4 shows the relationship, through simple linear regression analysis, between continuous predictors and procedural time. These unadjusted estimates show the average increase or decrease of procedural time per unit increase in any of these predictors. Baseline hemoglobin and baseline bilirubin were significant predictors of procedural time. For every gram that baseline hemoglobin increased, the time of TIPS procedure decreased by an average of 6.1 minutes (p=0.052), and for each milligram that baseline bilirubin increased, the time of TIPS procedure increased by an average of 5.6 minutes (p=0.0007).

**Table 4:** Association among Demographic, Laboratory, Procedural, and Healthcare Provider Characteristics with the duration of TIPS Procedure. Simple Linear Regression Analysis (SLR), n =154. Abbreviations: SE: standard error, Kg: kilograms, cm: centimeters, mg: milligrams, mm: millimeters, d/L: deciliter, INR: international normalized ratio, AST: aspartate transaminase, ALT: alanine transaminase, ALP: alkaline phosphatase, PV: portal vein, PSGP: portosystemic pressure gradient, Staff Exp: staff experience, MELD: Model for End-stage Liver Disease, TIPS: transjugular intrahepatic portosystemic shunt

Variable	Number	Beta coefficient	SE	P value
Age	154	-0.78	0.53	0.15
Weight/Kg	143	0.16	0.27	0.5
Height/cm	141	-0.45	0.63	0.47
BMI kg/m^2	141	1.14	0.9	0.21
Hemoglobin gr/dL	153	-6.1	3.1	0.052
Creatinine mg/dL	153	-9.6	8.7	0.27
Sodium mg/dL	152	1.02	1.2	0.4
Bilirubin mg/dL	151	5.6	1.6	0.0007
INR	151	-9.7	20	0.6
AST mg/dL	150	0.03	0.02	0.3
ALT mg/dL	151	0.005	0.03	0.88
ALP mg/dL	151	0.04	0.08	0.58
Albumin gr/dL	147	-4.9	11.2	0.66
MELD	153	0.38	1.06	0.72
Diameter PV mm	87	-1.2	3.1	0.7
PSGP pre-TIPS	122	0.5	1.29	0.68
IR Experience. Years.	154	-1.73	1.19	0.15
Liters. Paracentesis	63	-4.6	3.6	0.2

Table 5 shows a final linear regression model with time as the outcome variable, and baseline bilirubin as the principal predictor. The data was stratified in categories according to the variable “Access”. The reason for stratifying the "Access" variable was because in the group of patients who required additional access or additional procedures, the time variation was not associated with the number of liver punctures; this is the case when intravascular ultrasound, splenic access or umbilical access was used. Stratification allows control for confounding, and interaction. This analysis produced one model for those patients who did not require additional access and another for those who required ‘additional access'. In the first model that corresponds to the subgroup of patients ‘No additional access’ the estimated increase in average time was 4.9 minutes per unit (mg/dl) increase in baseline bilirubin (p=0.005). The average procedural time was estimated to be longer for alcoholic cirrhosis (53 min, p=0.0002), NASH (43 min, p=0.02), and other types of cirrhosis (69 min, p=0.0002) compared to time in viral cirrhosis and after adjustment for bilirubin and staff experience. There was evidence of shorter procedural time among staff with two to four years of experience (-42 min, p=0.005), and staff with more than five years of experience (-42 min, p=0.002) compared to staff who had less than two years of experience after adjustment by bilirubin and type of cirrhosis (Table 5). In this model, the coefficient of determination R2 or the proportion of total variability of procedural time that the model explained was 0.29 (29%). In the model for patients requiring additional access, none of the predictors were associated with time.

**Table 5:** Linear Regression Models, Parameter Estimates on Procedural Time. Models Stratified by Access Abbreviations: Ref: reference, SE; standard error, IR exp.: interventional radiologist experience, NASH: non-alcoholic steatohepatitis

Model for ‘No additional access n=115’					Model for ‘additional accesses n=39’				
Variable	beta coefficient	SE	95%CI	P value	Variable	beta coefficient	SE	95%CI	P value
Intercept	134	13	107-161	< .0001>	intercept	252	51	148-357	< .0001>
Bilirubin mg/dl	4.9	1.7	1.5-8.3	0.005	Bilirubin mg/dl	5.5	3.2	-13	0.1
Viral cirrhosis	Ref.				Viral cirrhosis	Ref.			
Alcoholic cirrhosis	53	13	26 - 79	0.0002	Alcoholic cirrhosis	-68	36	5 - -142	0.07
NASH cirrhosis	43	19	6 - 81	0.02	NASH cirrhosis	-51	50	51 - -153	0.3
Other type	69	18	34 - 105	0.0002	Other type	-32	60	87 - -153	0.5
IR exp. <2 years	Ref.				Staff exp. <2 years	Ref.			
2 to 4 years	-42	15	-12 - -70	0.005	2 to 4 years	-16	51	88 - -119	0.8
5 or more years	-42	14	-15 - -69	0.002	5 or more years	-19	50	83 - -121	0.7

Stratification of the variable reason for TIPS produced two models, one for ascites and the other for GI bleeding (Table 6). After the adjustment, bilirubin was the only predictor of time in the GE bleeding group (Beta: 5 min, p=0.001).

**Table 6:** Linear Regression Models, Parameter Estimates on Procedural Time. Models stratified by reason for TIPS Abbreviations: Ref: reference, SE; Standard error, IR exp.: interventional radiologist experience, NASH: non-alcoholic steatohepatitis, TIPS: transjugular intrahepatic portosystemic shunt, GI: gastrointestinal

Model for ascites n = 76					Model for GI bleeding n = 78				
Variable	beta coefficient	SE	95%CI	P value	Variable	beta coefficient	SE	95%CI	P value
Intercept	145	19	108- 183	< .0001>	intercept	166	26	113-219	< .0001>
Bilirubin mg/dl	11.5	7.6	-4 - -26	0.13	Bilirubin mg/dl	5	1.9	1 - 9	0.001
Viral cirrhosis	Ref.				Viral cirrhosis	Ref.			
Alcoholic cirrhosis	19	18	-18 - 55	0.3	Alcoholic cirrhosis	26	24	-21 - 73	0.3
NASH cirrhosis	-1.5	23	-49 - 46	0.95	NASH cirrhosis	58	35	-12 - 129	0.1
Other type	36	26	-15 - 87	0.17	Other type	45	31	-17 - 108	0.15
IR exp. <2 years	Ref.				Staff exp. <2 years	Ref.			
2 to 4 years	-21	20	-63 - 19	0.3	2 to 4 years	-32	25	-82 - 18	0.2
5 or more years	-30	19	-70 - 8	0.12	5 or more years	-34	24	-83 - 114	0.2

When the subgroup of patients in ‘No additional accesses' (n=115) was divided in ascites and GI bleeding groups (stratification by “reason for TIPS”, the models of Table 7), baseline bilirubin, etiology of cirrhosis, and staff experience were predictors of the duration of the procedure in both models (ascites and GI bleeding). The effect of baseline bilirubin on the duration of the procedure was greater in the group of patients with ascites than in the group of patients with GE bleeding (19.5 minutes; p=0.006; vs 3.8 minutes; p=0.05; R2 = 34.5%).

**Table 7:** Linear regression models in patients that did not require additional access (n=115) stratified by reason for TIPS, Parameter Estimates on Procedural Time Abbreviations: Ref: reference, SE; standard error, IR exp.: interventional radiologist experience, NASH: non-alcoholic steatohepatitis, TIPS: transjugular intrahepatic portosystemic shunt, GE: gastroesophageal

Model for ascites n = 57					Model for GE bleeding n = 58				
Variable	beta coefficient	SE	95%CI	P value	Variable	beta coefficient	SE	95%CI	P value
Intercept	120	17	87-154	< .0001>	Intercept	133	23	87- 178	< .0001>
Bilirubin mg/dl	19.5	6.7	6 - 33	0.006	Bilirubin mg/dl	3.8	1.9	0 - 7.7	0.05
Viral cirrhosis	Ref.				Viral cirrhosis	Ref.			
Alcoholic cirrhosis	47	16	16 - 79	0.004	Alcoholic cirrhosis	65	22	21 - 110	0.005
NASH cirrhosis	5.9	21.6	-37 - 49	0.8	NASH cirrhosis	103	31	41 - 166	0.002
Other type	49	25	0 - 98	0.05	Other type	88	27	34 - 141	0.002
IR exp. <2 years	Ref.				Staff exp. <2 years	Ref.			
2 to 4 years	-44	18	-81 - -7	0.02	2 to 4 years	-46	22	-90 - -2	0.04
5 or more years	-41	17	-75 - -6	0.02	5 or more years	-52	21	-95 - -10	0.02

There was no mediation effect or interaction between the main variable and the third independent variable considered to cause confounding or interaction in the models. When baseline hemoglobin was included in the multiple regression, it lost its statistical significance; thus, it was removed from the final model.

## Discussion

The findings of this research demonstrated a direct association between baseline bilirubin and duration of TIPS procedure in patients who did not require additional access. In summary, the multi-variable models for patients who did not require additional access showed that after adjusting by covariates, baseline bilirubin, type of cirrhosis, and staff experience remained statistically significant predictors of the duration of the TIPS procedure.

Bilirubin value before the TIPS procedure reflects the severity of liver dysfunction and is an independent predictor of 30-day mortality [11]. Liver fibrosis results from the perpetuation of the liver healing response. In a study that evaluated whether any correlation existed between liver function tests and stages of hepatic fibrosis, the total bilirubin value in fibrosis stage 4 was significantly higher compared to stage 0 (2.7 vs. 1.7, p<0.05) [12]. Additionally, significant liver stiffness reduction has been demonstrated, in advanced liver fibrosis in patients with chronic hepatitis C virus after direct-acting antiviral therapy [13]. Reducing stiffness in viral cirrhosis could facilitate hepatic puncture to create the shunt, thus reducing the duration of the procedure in this etiological group. Although no statistical differences were found, this study showed lower levels of bilirubin in patients with cirrhosis of viral etiology (1.6 mg/ml) and NASH (1.1 mg/ml) compared to levels of bilirubin in alcoholic cirrhosis (2.6 mg/ml) and other etiologies (2.6 mg/ml) (p=0.3). On the other hand, high baseline bilirubin levels could explain a longer duration of surgical time in patients with GI bleeding, where the average mean bilirubin values were 3.6 mg/Dl versus 1.6 mg/Dl in patients with ascites (p=0.004). The mechanism underlying the positive association between baseline bilirubin and procedural time could not be explained, but it is possibly associated with the different degrees of severity of liver fibrosis found in cirrhosis, decreased liver size, and modification of liver anatomy cause by cirrhosis, or unknown factors.

Physician experience is a factor that determines the likelihood of a successful outcome from surgery. Staff experience became a predictor of procedural time when introduced into the linear regression model for patients without additional access. When this subset of patients was stratified by reason for TIPS, health provider experience acquired statistical significance as a predictor of procedural time in the ascites and GE bleeding groups. In the 1980s, the experience curve described a negative relation between the volume of certain procedures in hospitals and the in-hospital post-operative mortality rate [14]. Likewise, in other surgical procedures, as the surgeon's experience increases, perioperative parameters such as operative time and length of hospitalization improve [15].

Limitations and strengths

The following limitations should be considered when interpreting results. First, this is a single-center and retrospective study, subject to accuracy, completeness, and type of available data. For example, the best metric to quantify the experience of the IR physician is the number of procedures performed before the intervention. However, the retrospective nature of the study made it impossible to obtain this information, limiting the study to using the years of interventional experience as the only metric available for this study. Second, this analysis did not account for variations related to intraoperator and interoperator skill differences. Third, the small sample size when the variables are categorized, as is the case with ethnicity, makes estimates for Hispanic + other ethnicities groups less consistent. Lastly, there was a group of patients with missing data, which decreased the sample size, limiting confidence intervals and p values. For these reasons, cautious conclusions should be drawn regarding the association between predictors and duration of procedural time. Moreover, the external validity or the degree to which the study findings can be extrapolated is limited by the patient, staff, and hospital characteristics. Some researchers considered a highly specialized center one that performs more than 50 TIPS procedures per year; in such centers, these results cannot be extrapolated. Some authors consider that an experienced operator can perform a TIPS in 30 to 120 minutes using conscious sedation [16-20]. In comparison, the Clinical Services and Standards Committee of the British Society of Gastroenterology recommends that service units that offer TIPS should perform a minimum of 10 cases per annum [20]. This study is the first that seeks to establish the predictors of duration of the TIPS procedure, including patient characteristics, staff experience, and type of hospital. Procedural time has been used to measure efficiency, quality, and cost-effectiveness in the operating room [21]. One of the study implications is to strengthen the experience of the IR physician, standardized the use of intravenous ultrasound, support staff, and other disciplines in the institution to ensure success and maximize the well-being of the patient who undergoes TIPS placement.

## Conclusions

Baseline bilirubin level, etiology of cirrhosis, and staff experience were the main predictors of the duration of the TIPS procedure in those patients who did not require additional access. The magnitude of the effect of baseline bilirubin on procedural time was higher in those patients with ascites when compared with the GI bleeding group. The mechanism underlying the positive association between baseline bilirubin and procedural time is possibly related to the degree of liver fibrosis.
